# Supplementary material for: An ECF-type transporter scavenges heme to overcome iron-limitation in Staphylococcus lugdunensis
Source: eLife. 2020 Jun 9;9:e57322. doi: 10.7554/eLife.57322 (PMC7299338; doi:10.7554/eLife.57322)
Supplement: Supplementary file 1. — PCR and qPCR primers sequences used in this study. [file elife-57322-supp1.docx]

| **Key Resources Table** | | | | |
| --- | --- | --- | --- | --- |
| **Reagent type (species) or resource** | **Designation** | **Source or reference** | **Identifiers** | **Additional information** |
| Sequence based reagent | lhaSTA_A | This study | PCR primer | Deletion of the *lhaSTA* locus AGGGAACAAAAGCTGGGTACCGCAGGTGTATTATTTTCTGATG |
| Sequence based reagent | lhaSTA_B | This study | PCR primer | Deletion of the *lhaSTA* locus CATTTGTCACACTCCTTTAATG |
| Sequence based reagent | lhaSTA_C | This study | PCR primer | Deletion of the *lhaSTA* locus TTAAAGGAGTGTGACAAATGTAAATAAATATTAAATAAGACG |
| Sequence based reagent | lhaSTA_D | This study | PCR primer | Deletion of the *lhaSTA* locus CTATAGGGCGAATTGGAGCTCCACCAAAAGTAGTTCAACCTGC |
| Sequence based reagent | lhaSTA_ScF | This study | PCR primer | Screening of the *lhaSTA* deletion TTGTAGTTGTAGCTACTGTCTTG |
| Sequence based reagent | lhaSTA_ScR | This study | PCR primer | Screening of the *lhaSTA* deletion CACCTGTACCTGAATTAACTACAG |
| Sequence based reagent | isdEFL_A | This study | PCR primer | Deletion of the *IsdEFL* locus AGGGAACAAAAGCTGGGTACCGAAAAACGTAACAAAGATAAAC |
| Sequence based reagent | isdEFL_B | This study | PCR primer | Deletion of the *IsdEFL* locus CATTTTGTTACTCACCGCTTTC |
| Sequence based reagent | isdEFL_C | This study | PCR primer | Deletion of the *IsdEFL* locus GCGGTGAGTAACAAAATGTGAAATTAGTGCTTCGATTATG |
| Sequence based reagent | isdEFL_D | This study | PCR primer | Deletion of the *IsdEFL* locus CTATAGGGCGAATTGGAGCTCGATATTTTGTATCGAATTGAATGC |
| Sequence based reagent | isdEFL_ScF | This study | PCR primer | Screening of the *IsdEFL* deletion GCTAGGTGTAAAACATCCAAATG |
| Sequence based reagent | isdEFL_ScR | This study | PCR primer | Screening of the *IsdEFL* deletion CTTTCGTCGTTGTTTGATAAGC |
| Sequence based reagent | qPCR 5srRNA_F | This study | PCR primer | RT-qPCR quantification of *5srRNA* expression GCAAGGAGGTCACACCTGTT |
| Sequence based reagent | qPCR 5srRNAR_R | This study | PCR primer | RT-qPCR quantification of *5srRNA* expression GCCTGGCAACGTCCTACTCT |
| Sequence based reagent | qPCR lhaS_F | This study | PCR primer | RT-qPCR quantification of *lhaS* expression |
| Sequence based reagent | qPCR lhaS_R | This study | PCR primer | RT-qPCR quantification of *lhaS* expression |
| Sequence based reagent | qPCR lhaA_F | This study | PCR primer | RT-qPCR quantification of *lhaA* expression AGCATTATCTGGTGGGCAAC |
| Sequence based reagent | qPCR lhaA_R | This study | PCR primer | RT-qPCR quantification of *lhaA* expression TTCATCCGTACAAGCCATCA |
| Sequence based reagent | lhaS_F | This study | PCR primer | His-tagged expression of LhaS using pQE30  ATTAAAGGAGCATGCCAAATGAAGAGAC |
| Sequence based reagent | lhaS_R | This study | PCR primer | His tagged expression of LhaS using pQE30 ACCTAAGCTTTTAAATCATACCTGCACGT |
| Sequence based reagent | Lha complement-F | This study | PCR primer | Expression of LhaSTA using pRB473 CGTATTGAAGGATCCTGATTTGG |
| Sequence based reagent | Lha complement-R | This study | PCR primer | Expression of LhaSTA using pRB473 CTCAACAGAAAACTGAGATTTCGTCTTATTTAAGCTTTATTTA |
